# A Canadian-wide perspective on the essential conditions for taking a comprehensive school health approach

**DOI:** 10.1186/s12889-020-09987-6

**Published:** 2020-12-14

**Authors:** Kacey C. Neely, Genevieve R. Montemurro, Kate E. Storey

**Affiliations:** 1 Faculty of Health Sciences and Sport, J12 Pathfoot, University of Stirling, Stirling, FK9 4LA UK; 2 School of Public Health, University of Alberta, 3-50 University Terrace, 8303-112 Street, Edmonton, AB T6G 2T4 Canada

**Keywords:** Canada, Comprehensive school health, Essential conditions, Health promotion, Qualitative research

## Abstract

**Background:**

The primary purpose of this research was to explore Comprehensive School Health (CSH) stakeholders’ perceptions of the essential conditions for taking a CSH approach in other contexts across Canada. The secondary purpose was to examine the need for and development of an evaluative tool or resource to assess the implementation of the essential conditions.

**Methods:**

Data were generated through individual semi-structured interviews (*n* = 38) and small group interviews (*n* = 3) with 45 participants across Canada involved in implementing policies or programs which take a CSH approach. Interviews were subjected to content analysis.

**Results:**

There was positive support for the essential conditions and results indicated the essential conditions are relevant across Canada. Findings revealed the necessity for a new essential condition that reflected support and leadership from the school district and/or provincial/territorial governing bodies. Modifications to the description of each of the essential conditions were also suggested to provide clarity. Results also indicated that an evaluative tool that was concise, meaningful, and provided immediate feedback would be useful to school communities to establish readiness, assess, and improve ongoing implementation of CSH approaches.

**Conclusions:**

This research contributes to the evidence-base of CSH by providing school communities across Canada with a set of refined and understandable essential conditions that support successful implementation. Further, the development of an evaluation tool will support school health champions, researchers, and policymakers in the optimization and implementation of policies or programs which take a CSH approach, ultimately supporting healthier school communities across the country.

## Background

Comprehensive School Health (CSH) is an internationally recognized framework that holistically addresses school health by transforming the culture of the school, incorporating individual, interpersonal, community and organizational factors [1]. School jurisdictions across Canada, and internationally, are adopting the CSH approach or its’ equivalencies (e.g., Health Promoting Schools, the Whole School, Whole Community, Whole Child Model) [2, 3]. Research has demonstrated the effectiveness of taking such comprehensive approaches to support the creation of health-enhancing behaviors among students [4–6], with mixed, but promising evidence for the improvement of educational and other outcomes [5, 7–10]. Specifically, a recent systematic review [5] found evidence for the effectiveness of the health promoting schools framework on body mass index, physical activity and fitness, fruit and vegetable consumption, tobacco use, and reports of being bullied. This study found there was insufficient evidence related to academic outcomes and emphasized the need for further research in this area.

While the body of evidence related to CSH outcomes continues to advance to address existing gaps in the knowledge base [8], factors related to CSH implementation remain an area of focus. Specifically, there is a need to focus on how CSH is implemented within school communities, with increased attention on identifying the conditions necessary for success. Helping school communities to recognize areas of progress and challenge, including contextual factors that may accelerate or slow their efforts is of great benefit. Ongoing identification of the unique assets that exist within school communities, as well as areas requiring further attention can act to support the development and sustainability of changes among schools and school authorities [11, 12]. A defining feature of CSH is that it is responsive; school communities are able to tailor and adapt their individual approaches to the unique strengths and needs of their community, rather than following a prescriptive program or initiative. This flexibility, however, can prove challenging when applying traditional implementation evaluation concepts of fidelity, dose, and reach. As such, establishing common essential conditions necessary to support school communities taking a CSH approach is beneficial not only for implementation but also for assessment. Identification and agreement on these essential conditions is needed to enhance the specificity and rigor of current evaluation in this field.

In response to this dilemma, a recent secondary analysis of qualitative interview data collected among diverse school-community stakeholders taking a CSH approach in Alberta, Canada identified the essential conditions of CSH implementation [13]. Analysis revealed a number of core conditions (students as change agents, school-specific autonomy, demonstrated administrative leadership, dedicated champion to engage school staff, community support, evidence, professional development) and contextual conditions (time, funding and project support, readiness and prior community connectivity) that were essential for effective implementation. This research created a strong evidence-base and a set of conditions needed to shift school culture and improve child health when taking a CSH approach, however, it is not clear whether these conditions could be applied more broadly across Canada. Historically there has been varied investment and capacity for school health across Canadian provinces and territories, resulting in a diversity of experiences, processes, and structures to support school communities taking a CSH approach [14, 15].

Increasingly, pan-Canadian organizations and groups are striving to align and collectively support CSH implementation in a coordinated way; building on the knowledge base and strong capacity that exists across regions. This is evidenced by the efforts of the Joint Consortium for School Health [1], established in 2005 and growing membership of the Canadian Healthy Schools Alliance, a collaboration of stakeholders in health and education with a goal to advance healthy school communities across Canada [16]. These collaborations aim to leverage existing knowledge and resources across disciplines and sectors, to promote and enhance provincial, territorial, and national efforts for CSH. Accordingly, there is increasing interest among school communities, school authorities, and health and education sectors on novel, user-friendly ways to meaningfully assess CSH implementation and measure progress. While a number of national and provincial toolkits exist to support schools to assess and improve their health promoting environments, often with a focus on specific health behaviours and related policies (e.g., the JCSH Healthy School Planner, the Alberta Healthy School Community Wellness Fund Handbook, WellSAT, CDC School Health Index, Maryland School Wellness Scorecard) [17–21], no resources are currently available that more broadly measure and evaluate successful CSH implementation. While useful and relevant to the CSH approach, existing tools are largely focused on specific health behaviours (predominantly healthy eating and physical activity) and policy implementation, rather than an assessment of more holistic shifts in school culture or conditions present to support CSH implementation processes. The increased focus and momentum to support the creation of healthy school communities presents an opportunity, and necessity, for increased coordination in the implementation and evaluation of CSH among school communities taking this approach. Therefore, the purpose of this research was two-fold: (1) To explore CSH stakeholders’ perceptions of the essential conditions for taking a CSH approach in other contexts across Canada, and (2) to examine the need for and development of an evaluative tool or resource to assess the implementation of the essential conditions.

## Method

Qualitative description methodology was used to address the purposes of this study. The qualitative description approach is best suited for research where a straight-forward description of a phenomenon is desired [22]. The goal of qualitative descriptive studies is a comprehensive summary of the specific event in everyday terms, staying close to the data and using the words of the individuals or groups of individuals involved. As such it was an appropriate approach to exploring perceptions of essential conditions for CSH and examining the development of a tool.

### Participants

Participants were purposefully sampled who could provide the ‘most’ and ‘best’ information to address the purposes of the study [23]. Individuals with experience implementing a CSH approach in a school community in Canada, including school health champions, school staff, teachers, administrators, and individuals in organizations that support CSH implementation were eligible to participate. Individuals working in government, including the ministries of health or education, were also included. Participants were identified and recruited through two main strategies. In the first strategy, members of a national body comprising 25 provincial, territorial, and federal ministries of health and education working to promote CSH were emailed the study information letter. The information letter provided a description of the study, and invited them to contact the lead researcher if they wished to participate. Of the 25 potential participants contacted, 8 participated in an interview and took part in the study. The second recruitment strategy was snowball sampling [24], whereby participants were asked to share the details of the study with stakeholders, colleagues, and other individuals they knew who would be able to speak to the essential conditions and CSH. This second strategy resulted in 37 more participants being recruited for the study. Once email contact was made, an interview or focus group was scheduled. Prior to the interview, participants reviewed the information letter with the lead researcher and provided verbal informed consent, as per research ethics approval from the University Research Ethics Board.

Participants were 45 individuals (31 females, 14 males; *M* age = 45.1 years, SD = 10.17) who were involved in CSH across Canada. Participants included individuals who were school health facilitators, teachers, and administrators (*n* = 10), program/organization managers and directors (*n* = 18), school health consultants (*n* = 6), and individuals involved in CSH within the health or education ministries (*n* = 11). In total, 19 participants represented the health sector, 23 represented the education sector, and three represented both health and education sectors. All Canadian provinces and territories with the exception of Nunavut were represented. Seventeen participants represented the Western provinces, 13 represented the Central provinces, nine represented the Eastern provinces, three represented the Territories, and there were three representatives from national organizations. All participants had a post-secondary undergraduate degree, 21 had a master’s degree, and five had a doctorate degree.

### Data generation

Data were generated through individual semi-structured interviews (*n* = 38) and small group interviews (*n* = 3). Both individual and small group interviews were used to generate data to accommodate participants. The small group interviews were preferred by stakeholders who worked as teams in the area of CSH. All interviews were conducted by the first author and were audio-recorded. Interviews lasted, on average, 50 min (*SD* = 11.25, range = 35–76). One week before the interview, participants were e-mailed a copy of the original essential conditions manuscript [13], an infographic summarizing the essential conditions, and a copy of the interview guide. They were asked to think about their experience working in CSH and reflect on the essential conditions to help enable them to provide thoughtful responses during the interview. The interview guide which was developed by the researchers (see suppl 1), was based on the essential conditions for implementing CSH [13] and qualitative interviewing guidelines by Rubin and Rubin [25]. The interview guide included introductory, main, and summary questions, starting with general questions and becoming more specific as the interview progressed. Probes and follow-up questions were used throughout the interview to maintain the flow of conversation and encourage participants to expand on their thoughts and ideas, which provided clarity and depth about their perspectives on the essential conditions and experiences with implementing CSH [25].

Introductory questions were used to gain demographic information and develop rapport with participants. The main questions were broad and open-ended and focused around two main research questions. The first set of questions were about the essential conditions for successful implementation of CSH. For example, participants were asked their perception on whether the currently identified conditions were comprehensive enough, whether they sufficiently captured the elements necessary for successful CSH implementation, and whether any additional elements needed to be added. The second section of the interview guide asked questions regarding the development of a tool or resource to evaluate CSH implementation based on the essential conditions. For example, participants were asked if they thought a tool or resource would be useful, and if so, what form they thought it should take. Finally, participants were asked summary questions to further reflect on their thoughts on and experiences with CSH and its implementation in Canada. Summary questions also provided participants with an opportunity to discuss any other aspects not covered by the main questions.

### Data analysis

Audio-recordings from interviews were transcribed verbatim by a professional transcription service, which produced a total of 720 pages of single-spaced data (304,495 words). Participants were given a code (e.g., P1, P2) and all other identifying information (e.g., school name, program name) was removed to ensure anonymity. Transcripts were checked with audio recordings, and read and re-read thoroughly by the first author who conducted the data analysis. Data were then subjected to content analysis following the stages outlined by Miles and Huberman [26]. First, using a deductive approach, transcripts were broadly coded based on the essential conditions (core and contextual conditions) and tool development. However, we took care to avoid unduly forcing the existing conditions on the data, demonstrated by the fact that new conditions emerged (e.g., higher-level support). Once data were grouped by condition, data were inductively coded. The next step was looking for patterns and we identified common themes across the participants’ data that depicted the suggested modifications and additions to the existing essential conditions. At this stage, we also examined qualitative differences based on stakeholder groups but there were no evident differences in the data. Throughout this stage of analysis, a constant comparison approach was used which involved re-reading data in each theme to ensure they ‘fit.’ The last step involved writing the themes up in two parts; the changes to the essential conditions, and the themes related to the development of an evaluative tool. While the deductive logic was valuable in organizing the data themes around existing conditions, the inductive approach allowed existing conditions to be refined and new conditions generated.

### Methodological rigour

Strategies were embedded in the research design to help establish methodological rigour. The sampling strategies enabled us to recruit participants who could provide ‘information-rich’ accounts [23]. It was important to recruit participants involved in CSH at various levels, across all province and territories. Data collection and analysis occurred in an iterative process which allowed for self-correction during the study process and helped to establish when themes were adequately saturated and therefore, when data collection could end [27]. The concurrent data collection and analysis ensured we reached inductive thematic saturation before stopping data collection as no new codes or themes emerged thus no need to further interviews were needed. We clearly came to a stage where final participants were repeating responses, indicating data saturation [28]. The analytic steps were led by the first author during and following analysis. She engaged in regular discussions about emerging themes and patterns with the research team who acted as ‘critical friends.’ This team approach provided opportunities to critically reflect on the results, thus enhancing the trustworthiness of the study.

## Results

**Fig. 1:**
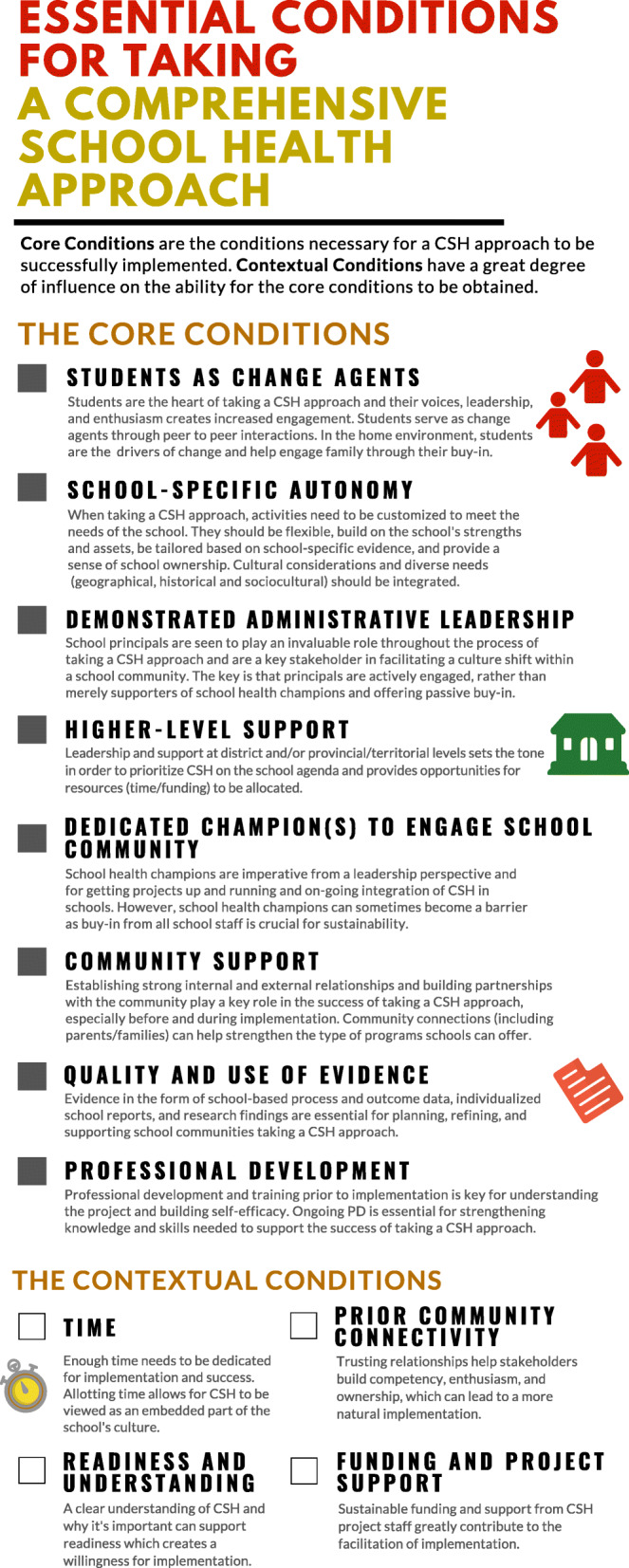
Essential conditions for taking a comprehensive school health approach

The results are presented in two parts to address the primary and secondary purposes of the research. First, we present a national perspective on the essential conditions and describe the modifications to the existing core and contextual conditions as well as present a new condition. Then we outline participants’ views on the need and format of an evaluative tool to assess the implementation of the essential conditions. Direct quotes from CSH stakeholders are used to support the results. Additionally, the modified essential conditions (in the form of an infographic) are summarized in Fig. 1.

### Overall perspectives on the essential conditions

Overall, participants from across Canada provided positive support for the essential conditions. The core and contextual conditions resonated with stakeholders involved in CSH at various levels. Participants expressed a general sense of gratitude that there was evidence to support what they had been doing in their school communities relating to CSH. For instance, Participant 20 said, “I just felt like oh my gosh, someone has actually captured our experience and in a way that we can understand it, and share it, and communicate it with our various organizations, or our community partners, or our school folks.” Further, participants felt that the essential conditions captured ‘what it takes’ to implement CSH and were practical guidelines for individuals working with staff and schools. Again, they particularly liked that the essential conditions provided evidence for implementation. One program director stated that “they’re easy to use, they’re easy to tailor, they’re easy to bring up as something that’s more tangible than just so-and-so’s word” (P31). Despite the variation across the country in terms of context, resources, and support available for CSH, the essential conditions were perceived as key factors that would support successful implementation of CSH.

Participants recognized the multiple layers of involvement required to successfully implement CSH and agreed with the breadth of the essential conditions. They highlighted a strength of the essential conditions was that it included all of the necessary people involved from students and teachers to the community and funders. Participant 12 explained that with the essential conditions “none are non-essential. Some will play a more important role at different times. I think in order to be truly comprehensive all those things need to sort of align in some way … It’s a complicated process.”

Many participants also noted the interconnectedness of the core conditions and contextual conditions. Specifically, participants noted that missing one condition could make the entire implementation process more challenging, thus highlighting the need for all conditions to be present. A participant involved in CSH at the ministry level explained how the “relationships between some of these conditions are absolutely contingent on others -- and not in a very easy to explain linear way -- but there’s certainly strong relationships between them” (P3). Multiple participants echoed these remarks regarding the intricate nature of the essential conditions. Participant 44 stated, “what is helpful about the core conditions and the contextual conditions idea is that they are simplifying an area that is very complex and nuanced.”

It is clear from the participants’ views that the original seven essential conditions were positively perceived. However, participants made several suggestions for modifications to improve the clarity and refine the conditions to best represent what is needed to implement CSH in school communities across the country (see Table 1 for a summary).

**Table 1:** Modifications to the essential core conditions

Theme	Modification	Representative Quote
Students as Change Agents	More emphasis on students’ voice	“I wonder about the student as change agent, the title is great, but it doesn’t, to me, it doesn’t get at student empowerment. I think if you can just tweak the language a tiny bit to bring out the student voice, so the students as leaders, so they are change agents” (P24).
	Clarify that students are change agents within the school through peer-to-peer interactions	“They [students] are much more likely to listen; they are much more likely to think about this [CSH] seriously when it’s coming from students from different grades” (P12).
School-specific Autonomy	Explicitly include school culture	“It’s probably a good idea to put a line in or a nuance in the school specific autonomy piece that recognizes cultural – various cultural influences in the demographic or something of the school” (P20).
Demonstrated Administrative Leadership	No modifications made	“The role of principals, I 100% agree” (P41).
Higher-level Support	*New essential condition:* Support and leadership from school district and government ministries	“The perfect situation is if you have leadership at the province and then you have leadership buying-in at the district level too … I think that having that district level support and that consistent, like not just a one-off, a consistent support and actually making room for it in the pile of many things, needs to be done” (P24).
	implementation of policy at the district and ministerial levels	“We’ve heard from the schools and from teachers time and again that it’s not going to happen, it’s not going to work until there is some kind of policy. And what I mean by that is until the Department of Education basically mandates that schools take this on, it’s not going to happen, which I didn’t see reflected in the essential conditions” (P1).
	Maintain balance between building accountability around CSH and school autonomy	“I think in some ways what we have right now in the CSH world or school health world is we have so much school-specific autonomy that we aren’t getting the systems change. So every school is doing their own thing and every school is doing something different. So I think there’s a really interesting balance there when we want to change things at a systems or a district level or a provincial level where we need to respect that autonomy” (P44).
Dedicate School Champion(s) to Engage School Community	Pluralize champion to reflect the team of champions that lead CSH	“I would definitely put champions – just make that plural” (P18).
	Engage the school community rather than just the school staff	“You need to have a champion pushing things within the community, and schools are communities so it would make sense that it’s community not just teachers” (P36).
Community Support	Broaden definition to include parents/families	“We say school community and hope that people realize it means home, school, and community broader than outside those four walls. So that might be a tweak, you know. I think that parents defined in there or families defined in there could strengthen it” (P31).
Quality and Use of Evidence	Collect data that is context-specific and meaningful to the school	“I think the big thing about evidence is that we need it to be local and meaningful. We need to give assistance in helping it be understood and just put into context and I think we are, we’re working harder to do that and to show places where the indicators and the core measurements are helping align with initiatives that are important to those who we are passing that data on to so that it becomes more meaningful to them. And then it becomes useful in a way by which therefore they want to monitor it and understand it (P4).
	Change definition to reflect the actual use of data	“We collected this data, now we have a survey, what are the results, what do they mean and what are we going to do about it are really challenging in school communities. The challenge is on the back end and actually using it. A lot of the data is out there, it’s just not being used” (P44).
Professional Development	No modifications made	“I would say that professional development needs to be a core condition. They’re not trained in this area of health necessarily. So they need that professional development to be able to fully understand why this [CSH] is important and buy in. To be those champions. To share it with their students, to share it with others in the school, to be able to speak the language and understand why the connection between being healthy and being able to learn” (P20).

### Modifications to the essential conditions

#### Students as change agents

Participants thought Students as Change Agents was essential for implementing CSH but felt the notion of students’ voices was left out of the description. Participants thought students’ voice needed to be included in the statement because students are at the heart of CSH and their voices give them a stronger sense of ownership and empowerment within the school community. Closely tied to student voice was student leadership. Like many stakeholders involved in CSH at all levels, Participant 7 stated, “change is definitely more profound if the youth have the leadership role and are supported by adults.”

The last modification suggested was to clarify that students are change agents within the school through peer-to-peer interactions, not just change agents to the home environment, which was the focus of the existing definition. Participants recognized the importance of parental buy-in and students’ roles in driving this change at home, but thought there should be more emphasis on being drivers of change at school. It was also evident that peer interaction drove change within school communities. Participant 23 said: “students have said to us before that they’re more likely to want to be involved in something or want to make changes when their peers are initiating it and are involved rather than adults telling them what to do.”

#### School-specific autonomy

All participants agreed that school-specific autonomy was “absolutely critical” (P5) for implementing CSH. One point raised by many stakeholders, particularly those engaged in CSH at the government level, was around school culture and cultural considerations and that this needed to be explicitly stated in the definition. For example, Participant 23 said, “You might have a word in there that includes like when you’re meeting the needs of the school, the culture, the socioeconomic make-up, like everything, identities of the students and things like that. I think that needs to be included there.” However, it was evident from participants who were involved in CSH at a school community level, that the notion of culture was already embedded in the autotomy of the school. In essence, these participants were already making geographical, historical, and sociocultural considerations. Participants also mentioned that regardless of the type of school (e.g., on-reserve schools, Catholic schools, French immersion schools, public schools) or geographic locale (e.g., rural, urban) CSH still ‘works’ because “that school-specific autonomy allows for that flexibility” (P31) and cultural considerations are part of making an individual school plan. Although most CSH stakeholders perceived cultural considerations to already be included in the definition of school-specific autonomy, the description has now been modified to explicitly include it.

#### Demonstrated administrative leadership

All participants fully supported the title and definition for the essential condition of demonstrated administrative leadership. No suggestions for modifications were made. From all levels of CSH involvement, stakeholder participants agreed, “if you don’t have the principals on-board you have nobody. You’re not getting in the door” (P29). They also thought that administrators needing to be actively engaged was an essential component of the description. Participant 25 explained, “I liked how you put in that the key of the principal, that they’re actively engaged, not merely coerced by the school champions and just passively saying, ‘yeah, do whatever you want’.”

#### New essential condition: Higher-level support

Stakeholders engaged in CSH strongly believed that an essential condition pertaining to higher-level support was missing. Participants, both at the government level and in school communities, discussed the need for support above the administrative leadership team. Participant 29 stressed the importance of higher-level support. She said, “You really have to have a buy-in and commitment to CSH at the policy decision making level because even if a principal agrees, if the board says ‘no we don’t have time for this’, then they have to say no” (P29). Participants felt that shared and coherent priorities across all levels (i.e., school district, and government ministries) were needed for CSH implementation to be successful.

In addition to leadership and support from multiple higher levels, several participants thought the implementation of policy at the district and ministerial levels around CSH was needed. With a policy in place, they thought there would be accountability for school boards and school communities to follow through with implementing CSH. However, participants were quite cognizant of the need to maintain balance between a top down and bottom up approach in order to safeguard schools’ autonomy while also building accountability around CSH. Participant 3 said, “it’s that combination of permission from the top that we’re going to do something and direction that this is important but also giving schools, at the ground level, all the flexibility to do it in a way that works for them” . Therefore, based on what CSH stakeholder participants reported, higher-level support was created as a new core condition.

#### Dedicated school champion(s) to engage school community

Participants suggested two small changes to this core condition, both of which related to the title. First, participants thought it should be pluralized to reflect the team of champions that often lead CSH in a school community. Participants also felt that teamwork was a helpful strategy for a CSH approach, especially in school communities where there was no health champion position and multiple individuals worked together. As Participant 20 indicated, “I think CSH is more successful when there is a team of champions.” The other change reflected in the title of this core condition is to engage the school community rather than just the school staff. It is important that the team of school health champions aim to engage all of those who are part of the school community including students, teachers, staff, parents, and the wider community.

#### Community support

Community support was an essential condition that participants in all contexts across Canada supported. It was perceived as crucial for CSH success and sustainability. Participants described community support and active engagement from public health, students, and parents “as the secret sauce. The more people that you’re involving in the conversations, the better this will be for everyone” (P39). While everyone agreed that community support was imperative and the title of the condition remained unchanged, participants felt the definition needed to be broadened to specifically identify parents and families because they play an important role in supporting CSH and may not always be included in ‘community’. This modification was clearly indicated by Participant 18 who stated, “parents are a critical piece … I put them [parents] under community support, but maybe you’ve ought to be more explicit on parents” .

#### Quality and use of evidence

All stakeholders mentioned that evidence was valuable in all stages of CSH. It was necessary for planning CSH in order to identify specific strengths and needs of the school community and was indispensable for implementation to assess what conditions were in place. Specifically, school-based evidence was key to evaluating CSH progress so changes could be made and new goals set, or simply put; planning, refining, and supporting implementation. One principal summarized the value of evidence in shaping CSH in her school. She said, “[evidence] has played a big role in what we do at times and other times it hasn’t played a big enough role. It waxes and wanes but it is the best way, I think, to know if something is working and also the best way to get ideas for the next step” (P10). Participants indicated the essential value of evidence to CSH, however, many participants also indicated that while data were available, it was not often used. Participant 32 was quite straight forward when admitting “yes there’s evidence, the problem with evidence is it’s not used.” The actual use of data that were readily collected and available appeared to be a challenge for school communities.

A further challenge with using data was that it is often collected on a provincial or territorial level rather than at the school level. As such, participants explained that evidence needed to be context-specific and be meaningful to the school community. Further, participants indicated that quality evidence meant that it was more than just survey data. Specially, they argued that some of the most meaningful data came from qualitative assessments with students. Based on these issues with evidence, the title of the essential condition has been modified to emphasize that data needs to be of meaningful quality, and once collected, it needs to be used in a manner that actually informs implementation.

#### Professional development

Participants thought professional development (PD) was an important aspect of implementing CSH, however, there were competing thoughts as to whether PD should remain a core condition or be moved to a contextual condition. Several participants felt PD would fit better as a contextual condition because PD was not perceived to be as necessary as some of the other core conditions like school-specific autonomy and administrative leadership. When asked why it should be moved to contextual conditions, Participant 15 said PD “is definitely helpful but I wouldn’t say that it’s as much of a priority as the other pieces.” Further, participants thought PD was not necessarily required to implement CSH and saw it more of a ‘bonus’ for teachers. One the contrary, several participants believed PD was essential to the success of implementation and should stay a core condition. As Participant 13 commented, “PD is absolutely critical for promoting school health. We’re not all wellness experts on our own.” They thought on-going training and education on what CSH is and why it is valuable was important to develop administrators’, teachers’, and school board staff’s knowledge and skills around CSH and build their capacity to do the work.

#### Contextual conditions

Overall, CSH stakeholder participants perceived the contextual conditions to accurately influence the ability of the core conditions to be met. They agreed with the contextual conditions of time, and funding and project support but felt it was necessary to have readiness and understanding and prior community connectivity as separate contextual conditions.

In terms of *time*, this contextual condition did not change. Participants reported that time significantly influenced the implementation of CSH. The biggest challenge was having the time to plan and implement, because without enough time, CSH implementation would not be done well or be successful. Participant 12 said that time was needed to “sit down, actually create some things. Having time to meet with students, have time to meet with other staff. And, of course, the time to bring parents in and community members to come be part of what’s happening in the school because, of course, everyone is busy; not just teachers.” Lastly, participants believed that time needed to be set aside for the multitude of tasks associated with implementing CSH.

*Funding and project support* was viewed as a significant resource for CSH implementation. This was indicated by one participant who said, “I think sometimes funding and supports really can make or break it” (P6). Funding and support was seen to influence almost all of the core conditions. It provides financial support for professional development such as release time for teachers, funds to support new initiatives or programs within a CSH approach and provides assistance to school health champions. Although a few participants suggested that funding and project support could be considered a core condition, Participant 24 said “I think those funding and project supports are contextual, they can be helpful, but if people want to do the work then they often find a way to do it.”

While they agreed with their importance, many participants thought *readiness and understanding and prior community connectivity* were unique and wondered why the two were combined as one contextual condition. “… why are those together? I think there’s too much to think about in a contextual condition. They should be separate ‘cause readiness alone is a pretty huge factor” (P2). Participants suggested separating ‘readiness and understanding’ and ‘prior community connectivity’ into their own contextual condition because on its own, they thought, “the readiness piece is huge, some schools just aren’t ready to be doing anything different than what they are” (P25). Readiness alone was seen as an imperative ‘first-step’ to engage in CSH and to change the school culture. One aspect of readiness that participants felt strongly about was having a shared understanding of CSH and how it connects to health and well-being in school communities. A teacher summarized the contextual condition of readiness and understanding best when she said, “if they don’t know about all this [CSH] stuff, how could they ever be ready? But I think they have to at least be ready or open to change” (P8). As such, the contextual condition of readiness and understanding describes the need for school communities to have a clear understanding of CSH and why it is important in supporting readiness for CSH implementation.

In support of the decision to separate these two contextual conditions, one participant stated, “I’d essentially think of them as two, kind of this idea of readiness to take on this approach and kind of embed this approach in what they’re doing, and then this idea of, you know, how do you start to engage the community in this work” (P19). Specifically, in relation to prior community connectivity, participants agreed that it could influence the core conditions, but perhaps not to the same extent as readiness, time, and support. One teacher said, “I don’t know that prior community connectivity is as big a piece but it definitely helps that they make those moves once they’re ready to go. It makes the things move faster it they have prior community connections” (P8).

### Tool development

Participants identified four key themes for the development of an evaluative tool. These included the format of the tool, the intent of the tool, identifying indicators, and using a team approach (see Table 2 for a summary).

**Table 2:** Development and format of evaluative tool themes

Theme	Description	Representative Quote
Format	The format of the tool needs to be user-friendly, concise, and available online.	“I think, to be honest, maybe the best way to do this is creating a tool that’s both able to be completed on paper as well as on like on an iPad or Smart Board or a computer, like digital, so that it can support wherever the school is at with their technology” (P34).
Intent	The tool should provide a clear idea of a school’s progress with implementation of the essential conditions.	“It has to be very easy to use, quick to use, has to get the information back to them [school health champions] as well in a way that they can use the information … It can’t just be sort of one-way information. It’d have to have a clear connection in terms of how is that tool supporting us and our schools to be able to be moving forward” (P3).
Indicators	CSH progress should be measured on a continuum with clear indication of what successful implementation looks like.	“You need that criterion so it’s outcome-based. What outcomes are you going to see if you’re at 100%? What outcomes are you going to see at 50%?...If I’m somebody that strives for excellence, I’m going to start looking at the criteria and say those are the outcomes that need to occur and then that’s what I’m going to strive for” (P30).
Team Approach	The tool should be designed in such a way that a team could complete it together.	“[The tool should be] a digital type thing that students and staff do together” (P37).

#### Format

Almost all participants believed a tool that could establish readiness and evaluate and assess the implementation of the essential conditions would be beneficial to school communities. One of the issues discussed by participants, which also closely related to the core condition of evidence, was that there was often not any reflection or evaluation of how a school was doing in terms of CSH implementation. It was clear from participants’ responses that there was a definite need for an evaluative tool, and participants across all levels of CSH stressed the importance of the format of the tool. Specifically, participants thought the development of an evaluative tool should be user-friendly and concise. For example, they said an evaluative tool “just needs to be quick and easy” (P20) and “something short and sweet” (P29). Participants also provided practical suggestions for the design and delivery of an evaluative tool. These included being available online and in paper format, as well as being available in multiple languages. Participant 40 described some of the key factors to consider:

It has to be online, it has to be in English and French, it has to be accessible in multiple languages. I think that it has to be really simple and it cannot be onerous. So I think that those would be some of your essential things that you need to be thinking about.

#### Intent

In addition to be a relatively short tool, participants also felt an evaluative tool should provide a clear idea of a school’s progress with implementation of the essential conditions. Participant 21 said the tool should “provide accuracy but be fairly simple and not very time-consuming … If it captures snapshots of progress or successes, or gaps and failures too, like that would be incredible.” A key point made by participants was the notion that the tool would need to provide immediate information to schools related to each of the essential conditions. After completing the tool, the feedback would indicate where a school is at, and possibly provide resources and ideas to improve or continue with implementation success for each condition. As Participant 27 suggested, the tool would be “something that you would fill out and then it could give you maybe a breakdown of areas that need to then be worked on, and resources and tips for doing so. It’s just essential that there’s feedback right away that addresses some of those areas and gives ideas for how to do better.” Participants also indicated that these resources could be somewhat of an “idea bank” (P14) that would provide a list of examples and strategies other schools have used who have experienced success in implementation of the essential conditions. While the list would not be exhaustive, participants thought it would be useful if it could provide some novel ideas for schools.

#### Indicators

Participants suggested that progress should be measured on a continuum rather than a score or number. Different ideas were put forward but were similar in the sense that successful implementation of the essential conditions and creating a healthy school community was a process that took time. One participant said, “I’d really like if it gave you a snapshot of where you might be on a continuum, on a road to healthier school communities. I like the notion of kind of giving you some suggestions on tips on either how you continue to improve or what you might want to look at doing that you’re not doing” (P40). Likewise, Participant 23 provided a detailed description of what such a continuum could look like. She explained the ‘stages’ of implementation could be:

You know, ‘initial implementation’ or ‘initial stage’, then ‘further along’ [laughs] I don’t know what you would call it; and then like ‘fully implemented’. So some kind of scale like that that sort of says where they are at on the journey; or like ‘not at all’, like that might be the first one.

One necessary component of an evaluative tool that participants thought was important was indicators of successful implementation. This was especially important because there could be a lot of interpretation around what each essential condition could look like in practicality in a school. For example, several participants explained what student engagement looked like in one school community could be drastically different in another school community but both may think they have fully implemented the essential condition. Thus, having clear indicators for each essential condition would help in the creation of an evaluative tool.

#### Team approach

The last important consideration for developing an evaluative tool was that the tool be designed in such a way that a team could complete it. This aligns with the suggestion made earlier that the dedicated champion condition should be modified to dedicated champion(s) to engage school staff since a team approach is often the case when implementing CSH. Participant 2 said, “I think if you’re thinking of a tool, using a team approach is right. A tool that a team would use to evaluate where they’re at or an administrative team from a school to assess their stage of readiness.” Given successful implementation of the essential conditions for CSH encourages a team of dedicated health champions, the design of an evaluative tool should be made with this in mind.

## Discussion

The purposes of this study were to confirm the essential conditions for taking a CSH approach and whether they held true in other contexts across Canada. Additionally, we sought to determine if the development of an evaluative tool or resource was needed to assess the implementation of the essential conditions, and if yes, what form such a resource would take. Results indicated that the core and contextual essential conditions required for taking a CSH approach are comprehensive and relevant across provinces and territories in Canada. However, there was a need to incorporate a new condition: higher-level support. Further, participants suggested an evaluative tool that was short, web-based, and interactive with immediate feedback and strategies to enhance implementation would be beneficial to help school communities adopt a CSH approach. This is the first study to assess the essential conditions in a broader context and support that these core and contextual factors are necessary for success in changing school communities.

There was overwhelming support for the essential conditions. Despite historical resource differences for CSH among the ‘have’ and ‘have not’ provinces and territories in Canada [14, 15], results showed that the essential conditions held true regardless of location. This is an important finding given that the essential conditions were developed based on data from a province that was often described as well-resourced [13]. While some school jurisdictions, provinces, and territories may face several barriers because of a lack of resources available and competing demands, our findings demonstrate that even in these harder circumstances, the essential conditions still hold true. As reported by study participants, school-specific autonomy may be a particularly important condition for overcoming the potential disparities.

A modification to the ‘Students as Agents of Change’ condition was to be more explicit in emphasizing the role of student leadership and student voice in CSH, as well as their ability to influence others both within and outside of the school. While these modifications are minor, they provide additional depth to the critical role of students in CSH. McKernan et al. [29] found that student leadership, independence, and ownership helped them take a ‘take charge’ attitude in making healthy changes. Further, they found that students were leaders in driving positive changes in healthy eating and physical activity for themselves and their family members within the home. Evidently, students remain at the heart of CSH and these modifications to this essential condition amplify the student role. Participants in this study emphasized the importance of ‘Professional Development’, with some suggesting it could be situated as either a core or contextual condition. This underscores the importance of ongoing professional development and CSH-related learning, with varying levels of need throughout implementation and supports a clear link to a modified (and separate) contextual condition of ‘Readiness and Understanding’. For example, early learning opportunities that strengthen CSH knowledge and skills among school community members are necessary to establish implementation readiness, especially in contexts where knowledge is lacking. Ongoing, and more tailored professional development (e.g., learning how to use resources or promote specific health behaviours) can deepen implementation during later stages.

Existing research highlights the role of the principal in creating change and supporting the implementation of CSH [12, 30, 31]. Demonstrated administrative leadership was strongly supported by participants in this study, including the notion that active engagement, not just support, is crucial [13]. Beyond administrative leadership within the school, our findings indicated that leadership and support from higher levels is imperative. Hence, a new and necessary essential condition, ‘Higher-level Support’, became apparent. Participants at the government level and on the ground in school communities discussed the need for support above the administrative leadership team. Support from senior school authority leaders in school districts and school boards, as well as ministerial leaders in education and health gives autonomy to school leaders to pursue and promote CSH for their school communities. Higher-level support creates a culture where CSH is valued as part of the educational mandate of school communities.

Participants also discussed the potential need for policy to support school communities adopting a CSH approach. Research shows that comprehensive, and strongly and clearly written school authority wellness policies are more likely to be implemented and practiced effectively both at the district and school levels compared to policies that are weakly or vaguely written [32, 33]. Policies that are clear, but flexible, can promote accountability and lend credibility and direction to bolster implementation. As such, a balance between a top-down approach from school authorities, government and education/health ministries while maintaining school-specific autonomy is ever-so important. Further, a congruent national policy or strategy, coupled with established conditions for CSH may enable consistent monitoring and thus allow for a pan-Canadian perspective. One consideration then, is how a national CSH strategy and/or consistency in policy to support health promotion in schools across Canada, can increase alignment of cross-jurisdictional efforts and accelerate change. This would support systematic change and may be more likely to enhance the health and well-being of students, and whole school communities, across the country.

Further, this may be valuable in terms of coordination and support among ministries (and through bridging agencies) and can help to support what is happening at the school community level. Currently, school boards and governments, as well as other provincial and national groups are using competing frameworks and approaches to support and implement CSH. For real systematic change to take place, consistency and coherency in the way CSH is approached is necessary. The findings from this study demonstrate that stakeholders at varying levels and contexts all across Canada positively perceive the essential conditions for implementing CSH. Results also indicated that an evaluative tool that is concise, meaningful, and provides immediate feedback would be useful to school communities to establish readiness, assess, and improve ongoing implementation of CSH approaches. Development of a tool that is user-friendly and widely accessible for school communities across Canada can help promote alignment in our collective understanding of indicators of CSH success. This would directly address the lack of consistency in current measurement strategies and provide a set of common indicators and measures that are grounded in data from diverse communities across Canada. As an approach, CSH is an ongoing process that requires participation and engagement, and not an end goal achieved through a series of replicated steps. Because of this, defining common indicators and measures for processes that are inherently unique and participatory, has long presented challenges both in CSH and in health promotion evaluation more generally [34]. This research provides confirmation that the essential conditions for taking a CSH approach hold true across Canada. Thus, they provide a set of national CSH conditions that can be used as a foundation for the development of common indicators and measures to assess progress and build on our evidence-base of CSH. This is valuable to inform and advance CSH work in Canada, and internationally.

### Strengths & limitations

The main contribution of this study is the positive perception of essential conditions for taking a CSH approach that hold true across a range of contexts in Canada, thus providing a set of national conditions for CSH. A notable strength is the sample size and spread. We were able to recruit a large number of stakeholders from all across Canada involved in CSH at varying levels which enabled us to gain representative and multiple perspectives. However, despite this diversity, it is possible that additional perspectives were missed.

## Conclusion

This research provides momentum for coordination across the country to move CSH forward in an integrated and aligned manner. Our results indicate that the essential conditions are viewed by Canadian stakeholders as relevant and appropriate, with insights on how to tailor the format, intent, and indicators of a future evaluative tool to meet the needs of end-users. The current knowledge products (e.g., infographic, summary documents) have been updated to reflect the modified essential conditions and shared with stakeholders who are already using the essential conditions to support their practice. These include organizations, programs, and individuals across Canada. Based on the interest in an evaluative tool our next research stages will involve the development of this tool through stakeholder engagement and pilot testing, using principles of integrated knowledge translation [35]. Tool development will center on the needs outlined by stakeholders with input throughout tool creation and testing to enhance its’ relevance, clarity, and usability. To support CSH implementation, school communities saw value in a future evaluative tool that not only provides criteria for progress across conditions but is responsive to support implementation by supplying users with specific strategies and resources to overcome challenges. Early development stages will include a scan of existing assessment tools, with attention to sections and items that align with the essential conditions and implementation processes. For example, the WellSAT [19] includes specific implementation questions that focus on accountability, assessment, and engagement.

Overall this study contributes to the evidence-base of CSH implementation, and gives direction for future research to bolster the CSH framework. Given our findings, we believe there to be support and value in the widespread adoption of the essential conditions and we encourage pan-Canadian alignment on these conditions to support and advance CSH. To support this alignment, and to advance measurement of CSH, our future research will focus on the development and testing of an evaluative tool for school communities and school authorities, based on the essential conditions. The development of an evaluative tool will support school health champions, researchers, and policymakers in the optimization and implementation of policies or programs which take a CSH approach, ultimately supporting healthier school communities across the country.

## Supplementary Information

**Additional file 1:.** Suppl 1. Interview Guide. This file contains the interview questions participants were asked in the individual interviews and focus group interviews.

## Data Availability

The data used in the current study is available from the corresponding author on reasonable request and conditional HREB approval.

## Abbreviations

CSH: Comprehensive school health
JCSH: Joint Consortium for School Health
PD: Professional Development

## References

[1] Joint Consortium for School Health (2019). Comprehensive school health framework.

[2] World Health Organization (2016). Global school health initiative.

[3] Centres for Disease Control and Prevention. Whole School, Whole Community, Whole Child (WSCC) 2020 [updated February 10, 2020. Available from: https://www.cdc.gov/healthyschools/wscc/index.htm.

[4] Fung C, Kuhle S, Lu C, Purcell M, Schwartz M, Storey K (2012). From “best practice” to “next practice”: the effectiveness of school-based health promotion in improving healthy eating and physical activity and preventing childhood obesity. Int J Behav Nutr Phys Act.

[5] Langford R, Bonell C, Jones H, Pouliou T, Murphy S, Waters E (2015). The World Health Organization’s health promoting schools framework: a Cochrane systematic review and meta-analysis. BMC Public Health.

[6] Veugelers PJ, Fitzgerald AL (2005). Effectiveness of school programs in preventing childhood obesity: a multilevel comparison. Am J Public Health.

[7] Akiyama T, Njenga SM, Njomo DW, Takeuchi R, Kazama H, Mutua A, et al. Implementation of Kenyan comprehensive school health program: improvement and association with students’ academic attainment. Health Promot Int. 2020. 10.1093/heapro/daaa005. 10.1093/heapro/daaa005 32125374

[8] Langford R, Bonell C, Komro K, Murphy S, Magnus D, Waters E (2017). The health promoting schools framework: known unknowns and an agenda for future research. Health Educ Behav.

[9] Michael SL, Merlo CL, Basch CE, Wentzel KR, Wechsler H (2015). Critical connections: health and academics. J Sch Health.

[10] Murray NG, Low BJ, Hollis C, Cross AW, Davis SM (2007). Coordinated school health programs and academic achievement: a systematic review of the literature. J Sch Health.

[11] Deschesnes M, Martin C, Hill AJ (2003). Comprehensive approaches to school health promotion: how to achieve broader implementation?. Health Promot Int.

[12] Samdal O, Rowling L (2011). Theoretical and empirical base for implementation components of health-promoting schools. Health Educ.

[13] Storey KE, Montemurro G, Flynn J, Schwartz M, Wright E, Osler J (2016). Essential conditions for the implementation of comprehensive school health to achieve changes in school culture and improvements in health behaviors of students. BMC Public Health.

[14] Bassett-Gunter R, Yessis J, Manske S, Stockton L (2012). Healthy School Communities Concept Paper Ottawa, Ontario: Physical and Health Education Canada.

[15] Pan-Canadian Joint Consortium for School Health. Annual Report 2019 [updated September 30 2019. Available from: http://www.jcsh-cces.ca/wp-content/uploads/2019/11/JCSH_AR2019_EN.pdf.

[16] Physical and Health Education Canada. The Canadian Healthy Schools Alliance 2020 [Available from: https://phecanada.ca/activate/healthy-school-communities/canadian-healthy-school-alliance.

[17] Alberta Healthy School Community Wellness Fund (2015). Developing healthy school communities handbook.

[18] Joint Consortium or School Health (2013). JCSH healthy school planner: building healthier schools across Canada.

[19] Koriakin TA, McKee SL, Schwartz MB, Chafouleas SM (2020). Development of a comprehensive tool for school health policy evaluation: the WellSAT WSCC. J Sch Health.

[20] Maryland Public Schools. Maryland School Wellness Scorecard 2018 [updated March 2018. Available from: http://www.marylandpublicschools.org/programs/SchoolandCommunityNutrition/Documents/WellnessNutrition/MSDEWellnessScorecard.pdf.

[21] Centres for Disease Control and Prevention. School Health Index: a self-assessment and planning guide.: Centers for Disease Control and Prevention; 2000 [Available from: https://www.cdc.gov/healthyschools/shi/index.htm.

[22] Sandelowski M (2000). Whatever happened to qualitative description?. Res Nurs Health.

[23] Mayan MJ (2009). Essentials of qualitative inquiry.

[24] Patton MQ (2015). Qualitative research & evaluation methods.

[25] Rubin HJ, Rubin IS. Chapter 9: Designing main questions and probes. In: Qualitative Interviewing: The Art of Hearing Data. 3rd ed. Sage Publications; 2012.

[26] Miles MB, Huberman AM (2013). Qualitative data analysis.

[27] Morse JM (1995). The significance of saturation. Qual Health Res.

[28] Saunders B, Sim J, Kingstone T, Baker S, Waterfield J, Bartlam B (2018). Saturation in qualitative research: exploring its conceptualization and operationalization. Qual Quant.

[29] McKernan C, Montemurro G, Chahal H, Veugelers PJ, Gleddie D, Storey KE (2019). Translation of school-learned health behaviours into the home: student insights through photovoice. Can J Public Health.

[30] Lee JA, Welk GJ. Association between comprehensive school physical activity program implementation and principal support. Health Promot Pract. 2019. 10.1177/1524839919862767. 10.1177/1524839919862767 31315464

[31] Roberts E, McLeod N, Montemurro G, Veugelers PJ, Gleddie D, Storey KE (2016). Implementing comprehensive school health in Alberta, Canada: the principal’s role. Health Promot Int.

[32] Calvert HG, Turner L, Leider J, Piekarz-Porter E, Chriqui JF (2020). Comprehensive policies to support comprehensive practices: physical activity in elementary schools. J Phys Act Health.

[33] Schwartz MB, Henderson KE, Falbe J, Novak SA, Wharton CM, Long MW (2012). Strength and comprehensiveness of district school wellness policies predict policy implementation at the school level. J Sch Health.

[34] Nutbeam D (1998). Evaluating health promotion—Progress, Problems and solutions. Health Promot Int.

[35] Canadian Institutes of Health Research (2012). Guide to Knowledge Translation Planning at CIHR: Integrated and End-of-Grant Approaches. Ottawa, Ontarion, Canada.

