# Supplementary material for: A Canadian-wide perspective on the essential conditions for taking a comprehensive school health approach
Source: BMC Public Health. 2020 Dec 14;20:1907. doi: 10.1186/s12889-020-09987-6 (PMC7734767; doi:10.1186/s12889-020-09987-6)
Supplement: Supplementary file 1 — Additional file 1:. Suppl 1. Interview Guide. This file contains the interview questions participants were asked in the individual interviews and focus group interviews. [file 12889_2020_9987_MOESM1_ESM.docx]

**Interview and Focus Group Guide**

Before Starting…

- Quick introduction
- There are no right or wrong answers
- Gather demographic information

Demographic information:

| **Age** |  |
| --- | --- |
| **Gender** |  |
| **Position** |  |
| **Years ^** |  |

| **Other Positions (# years)** |  |
| --- | --- |
| **Level of Education** |  |
| **Degree** |  |

**Introductory Questions:**

1. Can you please describe your work/role in creating or promoting healthy school communities?

*Probes:*

*Where do you work?*

*What is the context/region?*

1. Have you read the Essential Conditions paper or looked over the Infographic? Any comments?
2. What are your overall thoughts/opinions on the essential conditions for implementation of comprehensive school health?

**Main Questions:**

1. Do you think these conditions sufficiently capture the elements necessary for successful CSH implementation? Yes or no? Please explain.
2. We’re going to go through each core condition now. Please explain why/how it is essential, or if you don’t believe it is, why not?
   1. Students as agents of change
   2. School-specific autonomy
   3. Demonstrated administrative leadership
   4. Dedicated champion to engage school staff
   5. Community support
   6. Evidence
   7. Professional development
3. What are your thoughts on the contextual conditions?

*Time*

*Funding and project supports*

*Readiness and prior community connectivity*

1. Are the essential conditions, as they are currently outlined, clear?
2. How do you think the definitions of these conditions are described? Are the definitions adequate? Please explain. *If not, what do you think is missing? Or how do you feel they could be improved?*
3. In your opinion, do you think any of the outlined conditions appear not to fit? / Do you think any are non-essential? *If so, which ones and why?*
4. Do you have any suggestions for any alternative condition that should go in its place or should be added?
5. Are there any regional conditions specific to (*insert region*) that you think we should consider?

**Tools for Knowledge Translation:**

1. We are wondering if the development of a resource or tool for using the essential conditions would be useful. How do you think this information should be provided? What would help support the optimization of CSH?

*Probes:*

*What type of format?
Implementation vs Evaluation?*

**Summary Questions:**

1. Where do you think your province/region/school board/school is at in terms of the essential conditions?
2. Aside from developing a tool specific for using the essential conditions, is there anything else you think would be beneficial?
3. Those are all the questions I have for you today. Is there anything I have missed or is there anything you would like to add?

Thank you for your time.
